# Quality of life and cost-effectiveness analysis of topical tranexamic acid and fibrin glue in femur fracture surgery

**DOI:** 10.1186/s12891-022-05775-y

**Published:** 2022-08-31

**Authors:** A Merchán-Galvis, M Posso, E Canovas, M Jordán, X Aguilera, MJ Martinez-Zapata

**Affiliations:** 1 Iberoamerican Cochrane Centre-Public Health and Clinical Epidemiology, IIBSant Pau, Barcelona, Spain; 2 Department of Social Medicine and Family Health, Universidad del Cauca, Popayán, Colombia; 3 Iberoamerican Cochrane Center - Biomedical Research Institute Sant Pau (IIB Sant Pau), Barcelona, Spain; 4 Department of Epidemiology and Evaluation, Hospital del Mar-IMIM, Barcelona, Spain; 5 Iberoamerican Cochrane Centre-IIB Sant Pau, Barcelona, Spain; 6 Orthopedic and Traumatology Service, Hospital de La Santa Creu I Sant Pau, Barcelona, Spain; 7 CIBERESP, Hospital de La Santa Creu I Sant Pau, Sant Antoni María Claret 165, Pavilion 18, 08025 Barcelona, Spain

**Keywords:** Quality of life, Hip fracture, Clinical trial, Cost-utility, Fibrin glue, Tranexamic acid

## Abstract

**Background:**

We assessed quality of life (QoL) of patients undergoing surgery for proximal femur fracture and performed a cost-effectiveness analysis of haemostatic drugs for reducing postoperative bleeding.

**Methods:**

We analysed data from an open, multicentre, parallel, randomized controlled clinical trial (RCT) that assessed the efficacy and safety of tranexamic acid (TXA group) and fibrin glue (FG group) administered topically prior to surgical closure, compared with usual haemostasis methods (control group).

For this study we conducted a cost-effectiveness analysis of these interventions from the Spanish Health System perspective, using a time horizon of 12 months. The cost was reported in $US purchasing power parity (USPPP). We calculated the incremental cost-effectiveness ratio (ICER) per QALY (quality-adjusted life-year).

**Results:**

We included 134 consecutive patients from February 2013 to March 2015: 42 patients in the TXA group, 46 in the FG group, and 46 in the control group. Before the fracture, EuroQol visual analogue scale (EQ-VAS) health questionnaire score was 68.6. During the 12 months post-surgery, the intragroup EQ-VAS improved, but without reaching pre-fracture values.

There were no differences between groups for EQ-VAS and EuroQol 5 dimensions 5 levels (EQ-5D-5L) health questionnaire score, nor in hospital stay costs or medical complication costs. Nevertheless, the cost of one FG treatment was significantly higher (399.1 $USPPP) than the cost of TXA (12.9 $USPPP) or usual haemostasis (0 $USPPP).

When comparing the cost-effectiveness of the interventions, FG was ruled out by simple dominance since it was more costly (13,314.7 $USPPP) than TXA (13,295.2 $USPPP) and less effective (utilities of 0.0532 vs. 0.0734, respectively). TXA compared to usual haemostasis had an ICER of 15,289.6 $USPPP per QALY).

**Conclusions:**

There were no significant differences between the intervention groups in terms of postoperative changes in QoL. However, topical TXA was more cost-effective than FG or usual haemostasis.

**Trial registration:**

ClinicalTrials.gov: NCT02150720. Date of registration 30/05/2014. Retrospectively registered.

## Background

Proximal femur fracture is one of the main causes of morbidity, disability, and mortality in the elderly population. In particular, patients with proximal femur fractures have an increased risk of mortality, which is also related to age, sex, dementia, and frailty, and can reach 18–33% per year [1].

The worldwide incidence of hip fractures is 14.2 million people and its prevalence is 23.6 million [2]. The age-standardized rate per 100,000 habitants for incidence is 182.5 and for prevalence is 298.1, compared with 1990 these numbers have increased by 0.4% and 7.5%, respectively [2]. Therefore, annually, one in every 1,000 people in developed countries will experience a hip fracture of the proximal femur and this incidence increases exponentially in people older than 80 years [2]. In Spain, there is an incidence of 104 cases per 100,000 inhabitants which means between 45,000 and 50,000 hip fractures per year; more than 90% will be treated surgically [3].

One of the main complications of surgical treatment is bleeding, which can cause anaemia. Hence, pharmacological and non-pharmacological therapeutic strategies are indicated to reduce bleeding, postoperative anaemia, and transfusions. Whereas tranexamic acid (TXA) and fibrin glue (FG) (a haemostatic product derived from fibrinogen) are drugs showing promising results, haemostasis using electrocautery is the most widely-used non-pharmacological intervention [4–6].

The resources needed to treat patients with proximal femur fracture are considerable and with a wide variation between high-income countries [7]. In a systematic review of the costs of hip fractures globally, the pooled estimate of the cost for the index hospitalization was $10,075 per patient [8].

Moreover, this amount can increase up to $43,669 per patientat the end of the first year of follow-up if health and social care costs are taken into account [8]. Therefore, it is important to identify and apply interventions that can reduce postoperative complications and hospital costs.

We previously conducted a randomized controlled clinical trial (RCT) that compared the safety and efficacy in reducing bleeding using topical TXA or FG versus the usual haemostasis technique in patients undergoing prosthetic hip replacement for proximal femur fracture [5, 9].

In the per protocol analysis, topical TXA showed a significant reduction in postoperative bleeding and transfusions compared with the alternatives. We hypothesized that topical TXA may also produce an improvement in QoL compared with the other therapeutic options and that it could be a saving option to implement in clinical practice. Therefore, we assessed the QoL of the included patients undergoing surgery for proximal femur fracture and performed a cost-effectiveness analysis of these treatments.

## Methods

We followed the CHEERS recommendations for reporting health-economics studies [10].

### Design and study population

An open, multicentre, parallel RCT was performed, including patients older than 18 years, with unilateral subcapital femoral fracture, treated with a total or partial hip prosthesis, who agreed to participate and gave signed informed consent [5]. The protocol was approved by the clinical research ethics committees of the participating hospitals, as well as by the Spanish Agency for Medicines and Health Products. The Spanish ethics committees (EC) that approved the clinical trial were EC of Hospital Universitari Germans Trias i Pujol de Badalona, EC of Hospital Universitari Mútua Terrassa, EC of Consorci Sanitari de Terrassa, EC of Fundació de Gestió Sanitaria Hospital de la Santa Creu i Sant Pau and, EC of Hospital Clinic Barcelona, Spain. The study was conducted following the guidelines of the Declaration of Helsinki.

The recruitment period was from February 2013 to March 2015 in six Spanish hospitals. Patients were excluded if they had a known allergy to TXA or FG, had multiple fractures or pathological fractures, a history of thromboembolic disease, ischaemic heart disease, thrombogenic arrhythmias or peripheral vascular disease, had a cardiovascular device, prothrombotic clotting abnormality, were on treatment with contraceptives or oestrogens, or those for whom informed consent could not be obtained prior to surgery [5].

After obtaining informed consent, 161 patients who met the inclusion criteria were randomly assigned to receive one of the study interventions with an allocation ratio of 1: 1: 1. The protocol was approved by the clinical research ethics committees of the participating hospitals, as well as by the Spanish Agency for Medicines and Health Products. The protocol number is registered in the database www.clinicaltrials.gov (NCT02150720). The study was conducted following the guidelines of the Declaration of Helsinki, and all patients or their legal representatives gave signed informed consent to participate in the study before surgery.

### Interventions

Control group: received haemostasis with conventional diathermy applied to the surgical wound surface before its closure.

TXA group: received TXA (Amchafibrin®, Rottafarm SL, Valencia, Spain), 1 g in 10 mL (concentration 100 mg/mL) of solution (2 vials of 500 mg/5 mL). Topical administration was performed through a special syringe that sprayed the product on the operative field before wound closure.

FG group: received 10 mL of FG (Evicel®, Omrix Biopharmaceuticals NV, Diegem, Belgium), which contains mainly fibrinogen, fibronectin, and human thrombin. FG was administered topically before wound closure using a special syringe supplied with the product. All researchers followed the instructions on the product data sheet.

### Outcomes

Results about efficacy and safety of assessed interventions were previously published [5]. Main outcome was blood loss collected postoperatively in the vacuum drains. Secondary outcomes were total blood loss, perioperative blood transfusions, units of transfused blood, postoperative complications, the length of hospital stay, treatment-related adverse events, and mortality.

### Health-related QoL

QoL was measured using the validated Spanish version of the generic questionnaire EQ-5D-5L (EuroQoL) [11].

Before surgery, patients were asked about their QoL prior to the fracture, which was considered the baseline value. We also assessed QoL after the fracture but before surgery, at 5 days post-surgery, and at 1-, 6-, and 12-months post-surgery. The patient interviews were conducted in person during the hospitalization period and by telephone after hospital discharge. When patients were unable to answer the questions about QoL, a family member or caregiver responded.

The EQ-5D-5L questionnaire assesses five dimensions: mobility, personal care, carrying out daily activities, pain/discomfort, and anxiety/depression. Each dimension has five severity levels (no problems, mild, moderate, severe, or extreme problems, or disability) with an ordinal score from 1 to 5. A score of 1 indicates a state of full health. In addition, this instrument performs a global assessment of the patient’s health through a vertical visual analogical scale (EQ-VAS) that ranges from 0 to 100 (best score). The patient is questioned about how his/her health has been on the day of the interview.

The partial results of the dimensions and the overall score of the EQ-5D-5L questionnaire were calculated according to the scoring algorithms developed by Ramos-Goñi et al. [12] QoL value for dead patients was considered ‘zero’ from the date of death until the end of follow-up at 12 months.

### Resources and costs

Total costs were calculated by multiplying the use of direct health care resources by their unit cost following the ‘bottom-up’ costing method. The bottom up method estimates unit costs at the service provider level using detailed data from records [13].

We included drug-related direct costs (TXA and FG) as well as medical care received during the hospitalization period. Hospitalization costs included the hospital stay, medical complications, complications related to the surgical wound, hematoma management, surgical reoperation, and units of blood received.

Unitary costs were obtained from the following data sources: a) the Financial Department of Hospital de la Santa Creu and Sant Pau, Barcelona, Spain; b) the database of the Spanish Hospital Costs Network (RECH, s.f.); and c) the scientific articles carried out in a similar context to our study [14, 15]. The cost of FG was obtained from a US study [16]. All costs were updated to the 2015-year value and were reported in the value of the US Dollar adjusted by purchasing power parity (USPPP) («PPP. Purchasing power converter», s.f.) (Table 1).

**Table 1:** Baseline characteristics of patients included in QoL assessment

	**Control** ***N* = 46** **n (%)**	**FG** ***N* = 46** **n (%)**	**Topical TXA** ***N* = 42** **n (%)**	***p***
Age (mean, SD)	84.06 (8.9)	81.74 (8.4)	83.57 (8.9)	0.412
Sex (women)	33 (71.7)	35 (76.1)	34 (81.0)	0.599
Medical background				
Hypertension	28 (60.9)	25 (54.3)	25 (59.5)	0.838
Surgery	19 (41.3)	20 (43.5)	20 (47.6)	0.493
Diabetes	11 (23.9)	11 (23.9)	5 (11.9)	0.287
Chronic obstructive pulmonary disease	2 (4.3)	8 (17.4)	5 (11.9)	0.128
Chronic renal disease	6 (13.0)	4 (8.7)	3 (7.1)	0.647
Digestive ulcer	1 (2.2)	2 (4.3)	0	-
Haematological disease	0	2 (4.3)	0	-
Chronic liver disease	2 (4.3)	0	0	-
ASA				
I	1 (2.2)	1 (2.2)	0	-
II	19 (41.3)	16 (34.8)	20 (47.6)	
III	18 (39.1)	9 (19.6)	13 (30.9)	
IV	1 (2.2)	2 (4.3)	0	
Unknown	7 (15.2)	18 (39.1)	9 (21.5)	
Surgery duration (Minutes, mean and SD)	77.9(24.33)	79.3 (23.53)	80.3 (20.3)	0.616
Prosthesis				
Monopolar	24 (52.2)	19 (43.2)	15 (35.7)	0.289
Bipolar	20 (43.5)	19 (43.2)	22 (52.4)	
Total	2 (4.3)	6 (13,6)	5 (11.9)	
Type of prosthesis				
Austin Moore®	24 (52.2)	23 (50.0)	17 (40.5)	0.638
HA Karey®	15 (32.6)	14 (30.4)	18 (42.9)	
Other	7 (15.2)	9 (19.6)	7 (16.6)	

*SD* Standard Deviation, *ASA* Scale of surgical risk assessment of the American Society of Anaesthesiologists, *TXA* Tranexamic Acid, *FG* Fibrin Glue

### Statistical analysis

#### Statistical analysis of QoL

Statistical analyses were performed according to a modified intention to treat approach, including all patients who were randomized, received the surgical intervention, and had assessments from at least two time points including a pre-surgical and post-surgical QoL. Patients with cognitive impairment were excluded from the analysis because the QoL assessment could not be obtained. The latest available data were assigned to missing data points.

Differences in proportion between categorical data were analysed using Pearson’s Chi squared test. For continuous data, we calculated mean and standard deviation (SD) or mean difference and 95% confidence interval (CI), and differences were assessed using parametric t-test assuming a normal distribution. For multiple comparisons we used one way analysis of variance (ANOVA) and the post-hoc Dunnett test.

We evaluated QoL throughout the study, from baseline to the end of follow-up. For changes in the VAS and EQ-5D-5L scores, a two-way ANOVA was performed. The factors were group (FG, TXA, and control), time (baseline, at day 5, and at 1, 6, and 12 months after surgery), and interaction between them. The ANOVA was performed using a general linear model (GLM) procedure.

Quality-adjusted life-years (QALYs) were calculated over the course of the 12 months using the area under the curve method based on the scores derived from the EQ-5D-5L questionnaire. Briefly, the QALY is a measure of the value of health outcomes that combines the length of life and the quality of this life into a single value (QALY) that can be compared across the three types of treatments. To calculate the amount of time patients are alive in a state of health, we used the date from the collection of the EQ-5D-5L value, up to the end of follow-up or death. To calculate the quality of this life, we used the utility values derived from the EQ-5D-5L associated with the state of health at day 5 and at 1, 6, and 12 months after surgery.

Death was assigned a utility of 0. The EQ-5D-5L utilities varied between 0 (worst state of health) and 1 (perfect health status). To calculate the QALY, the two measures are multiplied. For instance, one year lived in perfect health required a utility value of 1 at each time of evaluation (at day 5 and at 1, 6, and 12 months after surgery) and equates to 1 QALY. We summed the QALYs calculated at each of the evaluated times to obtain the utilities gained by one patient in one year. We then summed all QALYs gained in each treatment group to compare across treatment groups.

We used a two-sided significance level of 0.05. All statistical analyses were performed with IBM Corp. Released 2017. IBM SPSS Statistics for Windows, Version 25.0. Armonk, NY: IBM Corp.

### Cost-effectiveness analysis

This economic evaluation was developed from the Spanish Health System perspective. The time horizon of this study was 12 months. No discount rate was applied.

We first ranked the strategies by increasing cost. We used the definition of dominance provided by the York Health Economics Consortium: A dominant treatment option is one that is both less costly and results in better health outcomes than the comparator treatment (the former ‘dominates’ the latter). Conversely, a treatment option that is both more expensive and results in poorer health outcomes is said to be ‘dominated’ [17]. The strategy that was more costly and less effective than the preceding alternative was ruled out by simple dominance. Then, we calculated the incremental cost-effectiveness ratio (ICER), which is the ratio of the incremental cost difference (C_intervention_ – C_control_) and the difference in QALYs (QALY_intervention_ – QALY_control_) obtained with one intervention with respect to the control:

$$\mathrm{ICER}=({\mathrm C}_{\mathrm{intervention}}-{\mathrm C}_{\mathrm{control}})/({\mathrm{QALY}}_{\mathrm{intervention}}-{\mathrm{QALY}}_{\mathrm{control}}).$$

A deterministic sensitivity analysis was carried out to assess the effect of the variation of both the utilities obtained with topical TXA as well as its cost.

All the analyses were performed using the statistical package Excel, Redmond, Washington (2011).

## Results

### Characteristics of the study population

**Fig. 1:**
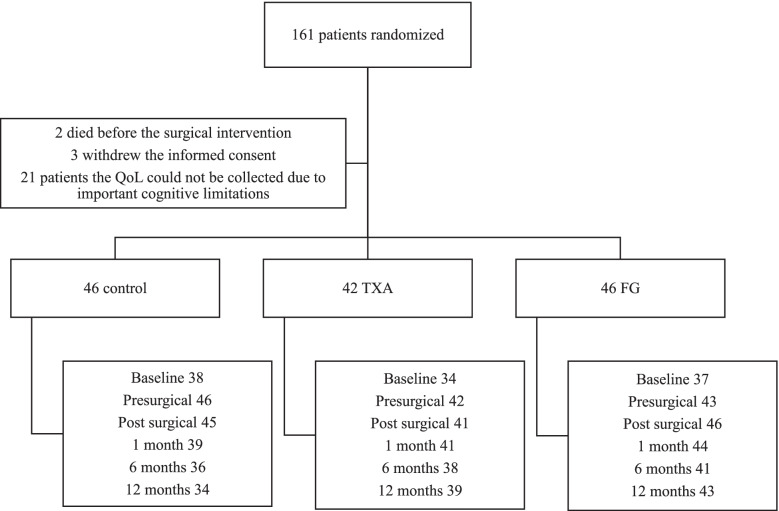
Study flow chart of patients that undergoing Quality of Life

One hundred and sixty-one patients were randomized. Two patients died before the surgical intervention, three patients withdrew their informed consent and in 21 patients the QoL could not be collected due to significant cognitive limitations. The number of exclusions was not statistically significantly different between the groups (*p* = 0.23). Forty-six patients were included in the haemostasis group (control), 42 in the topical TXA group and 46 in the FG group (Fig. 1).

Baseline characteristics of the population included for QoL assessment were similar between the study groups (Table 1). There were also no significant differences in hospital stay or medical complications (Table 2). During the 12-month follow-up, eight patients died in the control group, one in FG and one in TXA (*p* = 0.008).

**Table 2:** Outcomes related with cost

	**Control** ***n* = 46** **n (%)**	**FG** ***n* = 46** **n (%)**	**Topical TXA** ***n* = 42** **n (%)**	***P***
Hospital stays (mean days, SD)	11.3 (12.7)	11.3 (6.1)	11.6 (9.3)	0.987
Medical Complications				
Patients transfused	20 (43.5)	21 (45.7)	14 (33.3)	> 0.461
Number of red blood transfusions	37	44	25	0.272
Wound infection	3 (6.5)	0	2 (4.8)	0.076
Urinary tract infection/UAR	2 (4.3)	6 (13.0)	1 (2.4)	0.100
Cardiac or respiratory failure/ Respiratory Infection	1 (2.2)	2 (4.3)	0	0.387
Prosthesis complications	3 (6.5)	3 (6.5)	0	0.238
Surgical reintervention	4 (8.7)	2 (4.3)	5 (11.9)	0.467
Hematoma	3 (6.5)	7 (15.2)	2 (4.8)	0.381
Mortality	8 (17.4)	1 (2.2)	1 (2.4)	0.007

*TXA* Tranexamic Acid, *FG* Fibrin Glue

### Health-related QoL

The global assessment of QoL, measured using a VAS, was 68.6 at baseline (before fracture) with no statistically significant differences between groups (*p* = 0.517). After the fracture and prior to surgery, the global QoL assessment was 30.9, with no statistically significant differences between groups (*p* = 0.512).

**Fig. 2:**
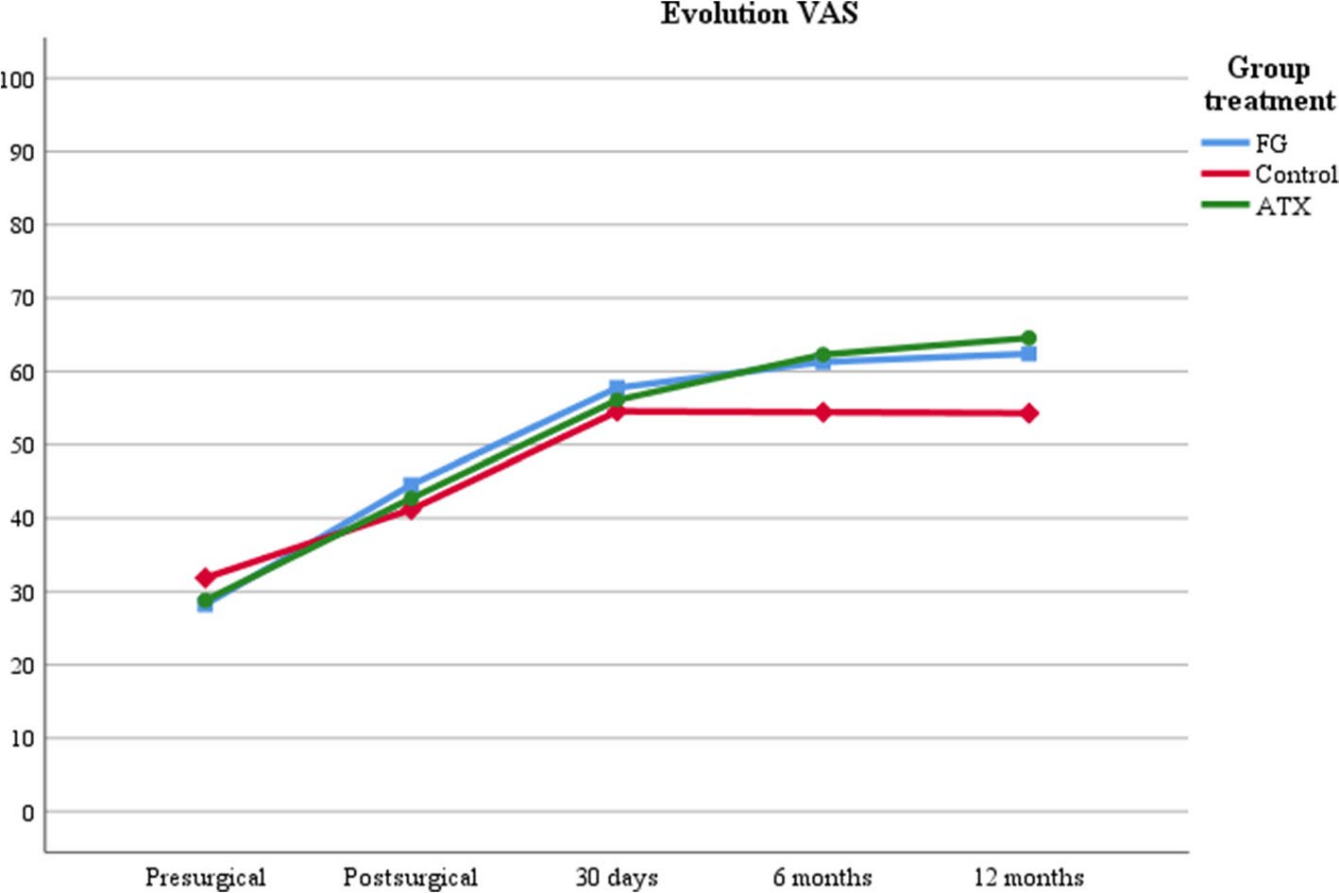
Evolution of QoL related to health for global visual analogue scale and by treatment. Notes: Multi-factor ANOVA. VAS evolution at post-surgery 5th day, 1-, 6- and 12-months follow-up: *p* < 0.001. Comparison between treatments at post-surgery 5th day, 1-, 6- and 12-months follow-up: *p* = 0.331

In successive follow-ups, there was a progressive improvement in QoL in all groups, but without reaching baseline values. QoL at 12 months was 63.1 (18.8) for TXA, 58.0 (19.9) for FG and 51.2 (26.9) for the control group (*p* = 0.045) (Table 3). VAS over the 12-month follow-up improved significantly within each group (*p* < 0.001), but there were no differences between groups (*p* = 0.331) (Fig. 2).

**Table 3:** QoL by haemostatic interventions of patients that underwent surgery after a femur fracture

	**Control** ***n* = 46** **mean (SD)**	**FG** ***n* = 46** **mean (SD)**	**Topical TXA** ***n* = 42** **mean (SD)**	**All patients** ***N* = 134** **mean (SD)**	***p***
**EQVAS**					
Baseline	66.3 (16.2)	69.4 (18.4)	70.6 (13.6)	68.6 (16.2)	0.517
Pre-surgical	32.1 (11.0)	31.3 (14.2)	29.1 (11.8)	30.9 (12.6)	0.512
Post-surgical	41.8 (16.8)	43.9 (15.3)	43.4 12.9)	43.0 (15.1)	0.782
1 month	54.4 (21.7)	56.2 (16.6)	55.9 (15.9)	55.5 (18.2)	0.889
6 months	52.9 (24.6)	58.6 (15.9)	61.7 (15.5)	57.7 (19.0)	0.980
12 months	51.2 (26.9)	58.0 (19.9)	63.1 (18.8)	57.1 (22.6)	0.045
**EQ5D5L index**					
Baseline	0.634 (0.229)	0.689 (0.317)	0.714 (0.268)	0.676 (0.272)	0.456
Pre-surgical	-0.390 (0.227)	-0.317 (0.323)	-0.447 (0.174)	-0.383 (0.255)	0.054
Post-surgical	0.104 (0.277)	0.221 (0.244)	0.139 (0.286)	0.155 (0.271)	0.103
1 month	0.355 (0.353)	0.443 (0.297)	0.400 (0.351)	0.399 (0.333)	0.459
6 months	0.442 (0.374)	0.533 (0.366)	0.593 (0.312)	0.520 (0.356)	0.133
12 months	0.435 (0.383)	0.529 (0.417)	0.626 (0.306)	0.527 (0.379)	0.059

*TXA* Tranexamic Acid, *FG* Fibrin Glue, *VAS* Visual Analogical Scale

**Fig. 3:**
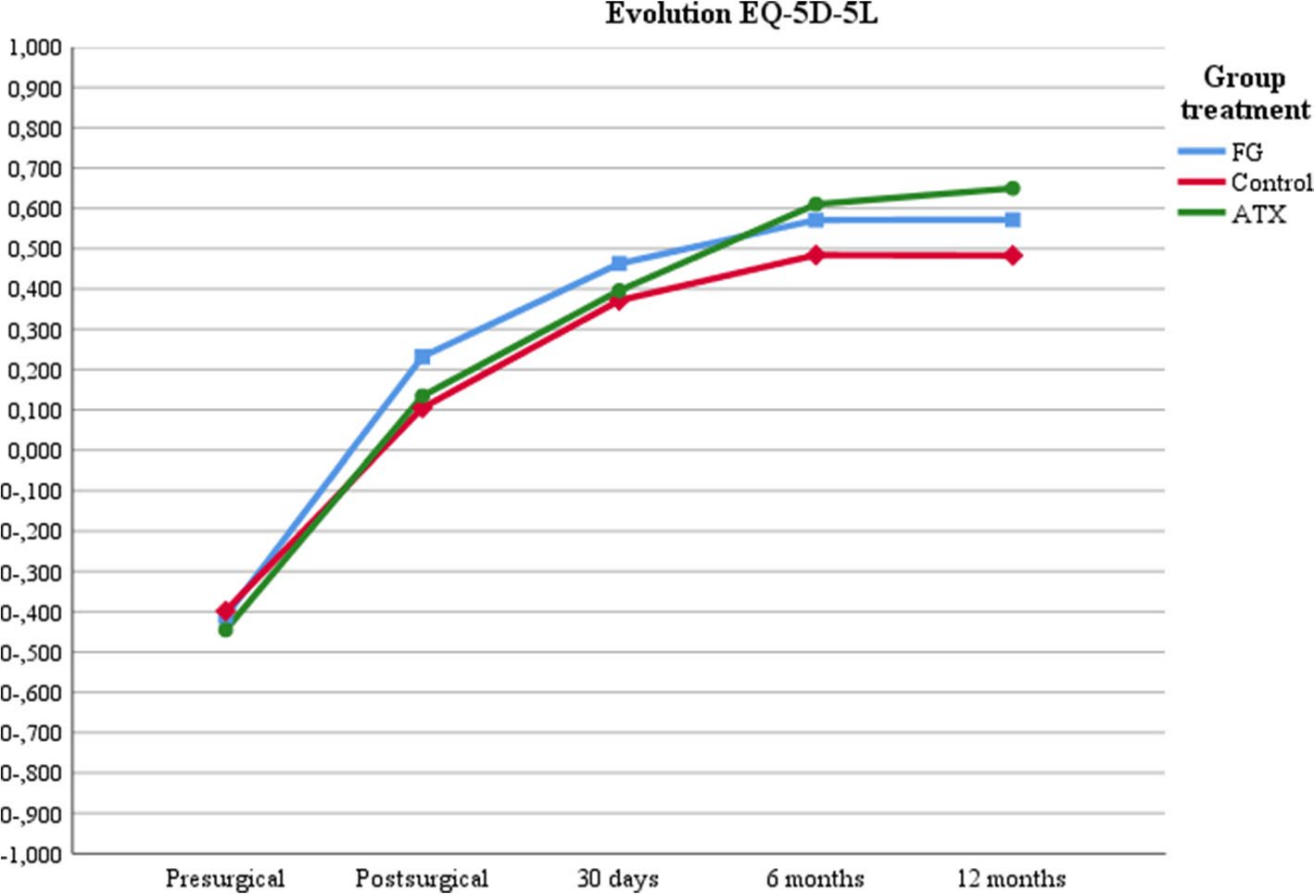
Evolution of QoL related to health for EQ5D5L and by treatment. Notes: Multi-factor ANOVA. EQ5D5L evolution at post-surgery 5th day, 1-, 6- and 12-months follow-up: *p* < 0.001. Comparison between treatments at post-surgery 5th day, 1-, 6- and 12-months follow-up: *p* = 0.121

The QoL index measured with the EQ-5D-5L instrument averaged 0.676 (0.272) at baseline (before fracture), was negative before surgery (-0.383) and improved after surgery, to 0.527 (0.379) at 12 months. There were no differences between groups in the QoL index (Table 3). EQ-5D-5L over the 12-month follow-up improved significantly within each group (*p* < 0.001) but there were no differences between groups (*p* = 0.121) (Fig. 3).

### Resources used and costs

We did not observe differences in hospital stay costs or in medical complications among groups (Table 4). However, the cost of one FG treatment was significantly higher than the cost of the TXA or usual haemostasis (399.1, 12.9, and 0 USPPP, respectively).

**Table 4:** Unitary costs used in the study, updated to 2015-year value, and reported in $USPPP

	**Source**	**Published cost**	**Country**	**Publication year**	**2015-year value**	**2015 USPPP**
Treatments						
Control	HSCSP	0.0	Spain	2015	0,0	0.0
Topical TXA	HSCSP	8.6 €	Spain	2015	8.6 €	12.9
FG	Corral 2016 [16]	399.1 $US	US	2016	399.1 $US	399.1
Hospital stays	HSCSP	601.2 €	Spain	2015	601.2 $	916.5
Medical complications						
Blood transfusion (unit cost)	Darbà 2009 [15]	350.0 €	Spain	2009	395.3 $	591.7
Wound infection	RECH	3191.0 €	Spain	2015	3191.0 $	4776.9
Urinary tract infection	RECH	1448.0 €	Spain	2015	1448.0 $	2167.7
Cardiac or respiratory failure	Allué 2014 [14]	11,107.0 €	Spain	2014	11,907.2 $	17,825.1
Prostesis complications	Allué 2014 [14]	7024.0 €	Spain	2014	7530.0 $	11,272.5
Surgical reintervention	RECH	9579.0 €	Spain	2015	9579.0 $	14,339.8
Hematoma	Allué 2014 [14]	3498.0 €	Spain	2010	3750.0 $	5613.8

*HSCSP* Financial Department of the Hospital de la Santa Creu and Sant Pau (Barcelona, Spain), *RECH* database of the Spanish Hospital Costs Network, *USPPP* $US Purchasing Power Parities are the rates of currency conversion that equalise the purchasing power of different currencies by eliminating the differences in price levels between countries, *TXA* Tranexamic Acid, *FG* Fibrin Glue, *€* Euros, *$* Dollar

The average cost per patient in the haemostasis group was 12,886 USPPP, versus 13,351 USPPP in the topical TXA group, and 13,483 USPPP in the FG group. Both the mean and median cost did not show significant differences among the groups (Table 5).

**Table 5:** Average costs in $USPPP according to the intervention group

	**Control *n* = 46**	**FG *n* = 46**	**Topical TXA *n* = 42**	**P**
Mean^a^	12,703.8	13,314.7	13,295.2	0.965
SD	14,483.8	8,802.3	13,372.1	
95%CI	8,518.2 – 16,889.4	10,770.9 -15.858,4	9,251.0–17,339.4	
Median^b^	9,431.8	10,671.4	10,094.3	0.188
Min	3,666.0	4,065.1	5,187.1	
Max	100,756.8	52,993.7	86,483.0	
Sum	584,374.0	612,474.3	558,399.6	

*TXA* Tranexamic Acid, *FG* Fibrin Glue, *USPPP* $US Purchasing Power Parities. ^a^ANOVA test, ^b^Median difference test

### Cost-effectiveness analysis

When comparing the cost-effectiveness of the interventions, FG was ruled out by simple dominance since it was more costly than TXA (13,314.7 USPPP vs. 13,295.2 USPPP, respectively), and less effective (0.0532 vs. 0.0734, respectively). TXA compared to usual haemostasis had an ICER of 15,289.6 USPPP per QALY (Table 6).

**Table 6:** Cost-utility analysis of control, topical TXA and FG for prevention of post-surgical bleeding

Treatment group	Costs ($USPPP)	Utilities (mean)	Incremental cost ($USPPP)	Incremental effect	ICUR
Control	12,703.8	0.029491	0.0	0,0	0
Topical TXA	13,295.2	0.073366	591.5	0.043875	13,480.3
FG	13,314.7	0.053200	19.4	-0.020200	Dominated

^*$*^*USPPP* $US Purchasing Power Parities are the rates of currency conversion that equalize the purchasing power of different currencies by eliminating the differences in price levels between countries, *ICUR* Incremental cost-utility ratio, *TXA* Tranexamic Acid, *FG* Fibrin Glue

### Deterministic sensitivity analysis

**Fig. 4:**
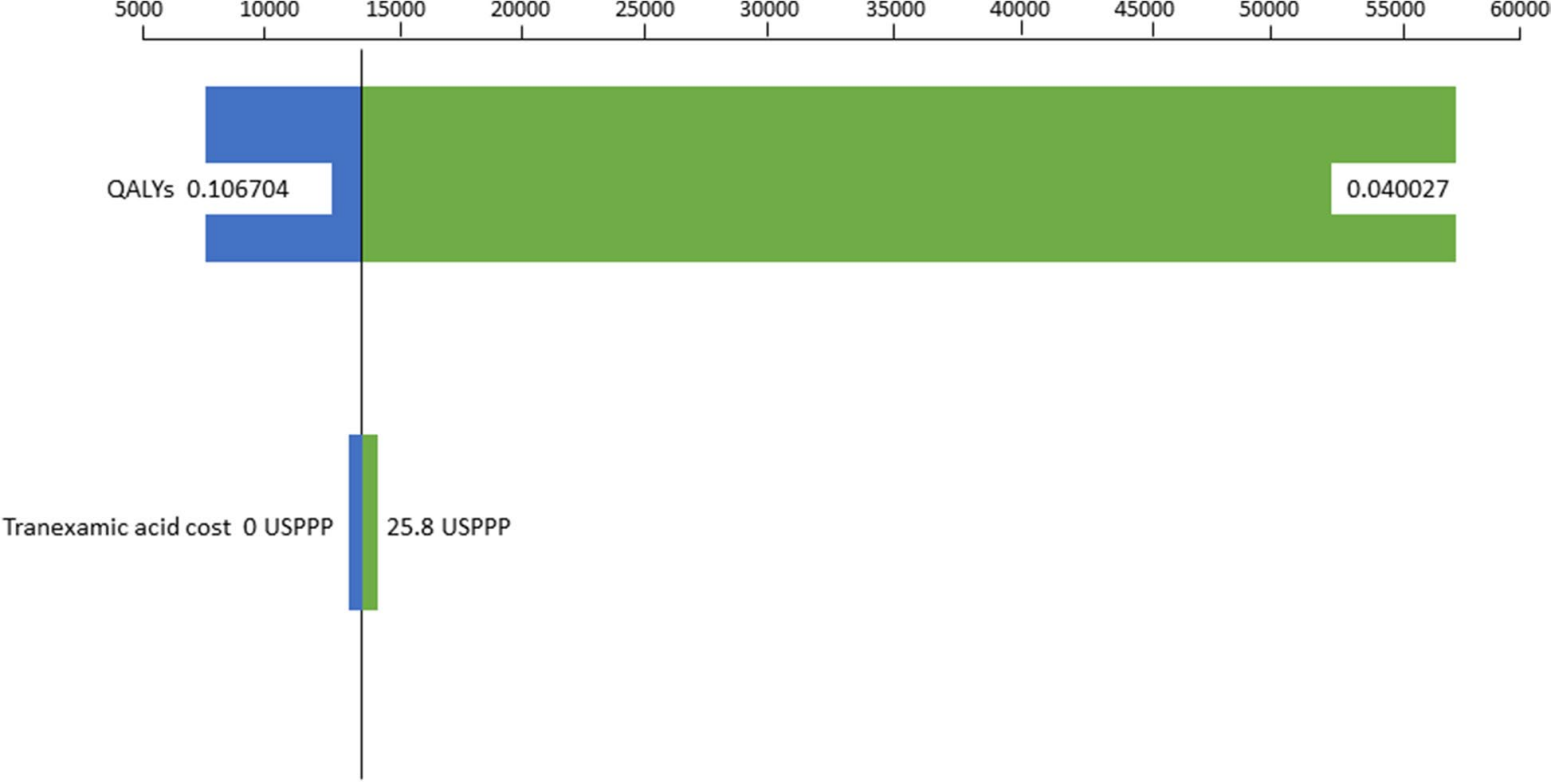
Sensitivity analysis of the incremental cost-utility ratio (ICUR). Notes: QALYs: Quality adjusted life years. The variation in QALYs was the 95% Confidence Interval upper (0.106704) and lower (0.040027) value of the mean utility (0.073366) in the topical TXA group. USPPP: $US Purchasing Power Parities are the rates of currency conversion that equalise the purchasing power of different currencies by eliminating the differences in price levels between countries. The variation in topical Tranexamic Acid (TXA) cost ranged from zero (control cost) and 25.8 USPPP, which is 100% higher than the unit cost of TXA

The deterministic sensitivity analysis was displayed in a tornado diagram (Fig. 4). Regarding the variation in TXA cost, this ranged from zero (cost of usual haemostasis) to 25.8 USPPP, which is 100% higher than the unit cost of TXA. For the variation in the topical TXA QALYs, we used the upper (0.106704) and lower (0.040027) value of the 95% CI of the mean observed QALYs (0.073366).

We found that the ICER was more sensitive to changes in TXA utilities than to its costs. An increase in utilities to a mean value of 0.1067 decreased the ICER to 7,660 USPPP, whereas when utilities decreased to a mean value of 0.0400, the ICER increased to 56,136 USPPP. In contrast, the effect of variation in TXA costs was less important. An increased cost of 25.8 USPPP increased the ICER to 13,774 USPPP, whereas the ICER was 13,186 USPPP when the cost of TXA was assumed to be zero.

## Discussion

We have presented the results on QoL and compared the cost-effectiveness of TXA, FG, and surgical diathermy for haemostasis in patients who underwent hip replacement surgery and agreed to participate in a randomized clinical trial that assessed the efficacy of these three interventions to reduce perioperative blood loss [5]. In our study, there were no significant differences in QoL and costs between treatment groups. However, the analysis from the perspective of the payer showed that FG proved to be a dominated intervention, being more expensive and offering fewer QALYs than topical TXA. Furthermore, we found that the ICER was more sensitive to changes in TXA utilities than its costs.

QoL improved after the surgical intervention but did not reach baseline pre-fracture values. These results are in line with other studies that demonstrated an improvement, especially in the first 6 months after the injury, without reaching pre-fracture value [18–21]. In our study, QALYs were slightly higher in the group treated with topical TXA both at six and 12 months of follow-up, but without reaching statistical significance.

Patients in the TXA group had less perioperative bleeding and transfusion requirements, and lower mortality, which, although not statistically significant, together would contribute to a greater overall patient recovery. The ICER calculated in our study was extremely sensitive to the profits obtained. In fact, in the sensitivity analysis, we observed that a small variation, coinciding with the 95% CI values of the average utility observed, can greatly affect the ICER.

Different studies have reported a reduction in direct care costs by decreasing costs associated with blood transfusion, laboratory testing, and length of stay when TXA is used, compared with the standard care. These studies showed an average saving of $US 331 [22] or € 284 [23] per patient when using TXA, which represents a 3-% reduction in hospital costs [22].

Based on our results, FG does not seem to be a recommendable intervention from the point of view of efficiency as it proved to be a dominated strategy, probably due to its cost.

Limitations of our study were the open design with a potential reporting bias and the sample size was calculated for the main outcomes of efficacy (blood loss), and not to evaluate differences in QALYs. Secondly, the information on unit cost did not come from a single source, which could affect the cost estimate. Another limitation was the lack of a probabilistic sensitivity analysis, which reduces the generalizability of the results to other contexts. The fact that we used the perspective of the payer and incorporated only the costs associated with the care received during hospitalization could also affect the accuracy of the estimation of the resources used. However, given that the study was randomized, it can be assumed that health care was similar in the three compared groups, and in fact, this was verified with the similarity of baseline characteristics.

The strengths of our study include the use of the validated EQ-5D-5L instrument to measure QoL and QALYs. In addition, we have not identified other studies that evaluate the cost-effectiveness of treatments to reduce post-surgical bleeding in our context. Some authors have calculated the direct costs of the drug, without considering the total cost of hospital medical care [24, 25]. One study analysed QoL and direct cost associated with the use of topical TXA in hip prosthesis [26], reporting a better QoL index in the topical TXA group than with placebo (0.715 vs. 0.686 respectively) but with follow-up limited to 3 months after surgery.

Likewise, another study analysed direct cost of the use of TXA in different presentations in patients with hip fracture [27]. This study reported the lowest cost when topical TXA was associated with intravenous TXA in comparison with placebo, but only bleeding and thromboembolic events were evaluated.

In short, patients with a proximal femur fracture, QoL improved after surgery without reaching baseline values at 12-months of follow-up. There were no significant differences in changes in QoL after surgery between the three interventions evaluated.

## Conclusion

Topical TXA can be an efficient intervention in patients with femur fractures. The application of this drug represents a similar or marginally higher cost and the possibility of obtaining a slight advantage in terms of QALYs when compared to usual haemostasis. FG would not be a first-choice treatment because it was a dominated strategy (i.e. more expensive and less effective). Future studies well-powered, multicentre, double-blind, or with assessor concealment and including patients reporting outcomes should confirm these results.

## Data Availability

The datasets generated and/or analysed during the current study are not publicly available due was not planned at the beginning of the study but are available from the corresponding author on reasonable request (Dr. Mª José Martínez-Zapata; e-mail: mmartinezz@santpau.cat).

## Abbreviations

QoL: Quality of life
TXA: Tranexamic acid
FG: Fibrin glue
US: United States
$: Dollars
£: Pounds Sterling
RCT: Randomized controlled clinical trial
USPPP: $US purchasing power parity
EQ-VAS: Visual analogical scale
SD: Standard deviation
CI: Confidence interval
ANOVA: Analysis of variance
QALYs: Quality-adjusted life-years
ICER: Incremental cost-effectiveness ratio
